# Pyocyanine

**Published:** 1861-07

**Authors:** 


PYOCYANINE.—The blue or green color of the pus of certain abscesses has been ascribed by different chemists to sulphuret of iron, ferrocyanuret of iron, or some other cyanogen compound; and M. Bergouhnioux contends that it is owing to the coloring matter of the bile. M. Fordos has, however, discovered the nature of this peculiar principle, and has given it the name of pyocyanine. It may be obtained in the following manner: the linen or bandages stained with the tinted pus are to be thoroughly soaked in water containing a little of the solution of ammonia, and a light bluish-green liquid is the result. To this is added chloroform, which extracts from the solution the blue principle, as well as the yellowish foreign matter. The chloroform is then drawn off, filtered, and evaporated, and distilled water is added to the residue, taking up the coloring principle, and leaving the fatty material. Chloroform is again admixed, and the solution treated as before. The blue principle and the yellowish matter being thus obtained, a few drops of chlorohydric acid are added, which redden the former, and the chloroform then dissolves the yellow material, leaving untouched the blue, which is rubbed up with carbonate of baryta, in order to get rid of the acid, and is, on the evaporation of the chloroformic solution, deposited in beautiful blue prismatic crystals. These are soluble in water, alcohol, ether, and chloroform; and the solution is reddened by acids, the natural color being restored on the addition of an alkali.

